# Supplementary material for: Evaluation of TiO2 Based Photocatalytic Treatment of Odor and Gaseous Emissions from Swine Manure with UV-A and UV-C
Source: Animals (Basel). 2021 Apr 30;11(5):1289. doi: 10.3390/ani11051289 (PMC8146479; doi:10.3390/ani11051289)
Supplement: Supplementary file 1 [file animals-11-01289-s001.zip › animals-1141416-supplementary.pdf]

animals

MDPI

Supplementary Material

Evaluation of TiO_2 based photocatalytic treatment of odor and gaseous emissions from swine manure with UV-A and UV-C

Myeongseong Lee [1], Jacek A. Koziel [1,*], Wyatt Murphy [1], William S. Jenks [2], Baitong Chen [1], Peiyang Li [1], Chumki Banik [1]

Supplementary Material 1. Generation of gaseous emissions from swine manure

The gas emissions generator system (Figure S1) was constructed using an air compressor (Dewalt, Baltimore, MD, USA), an air hose (1/4 in × 25 ft, Lowes, Mooresville, NC, USA). The compressed air (set at 5.4 atm, 80 psi) supply was connected to a circle with holes plated at the bottom of the manure. The circle was perforated with small holes for ~even size bubble generation, starting from 0.2 cm at the air inlet to 1 cm on the opposite side.

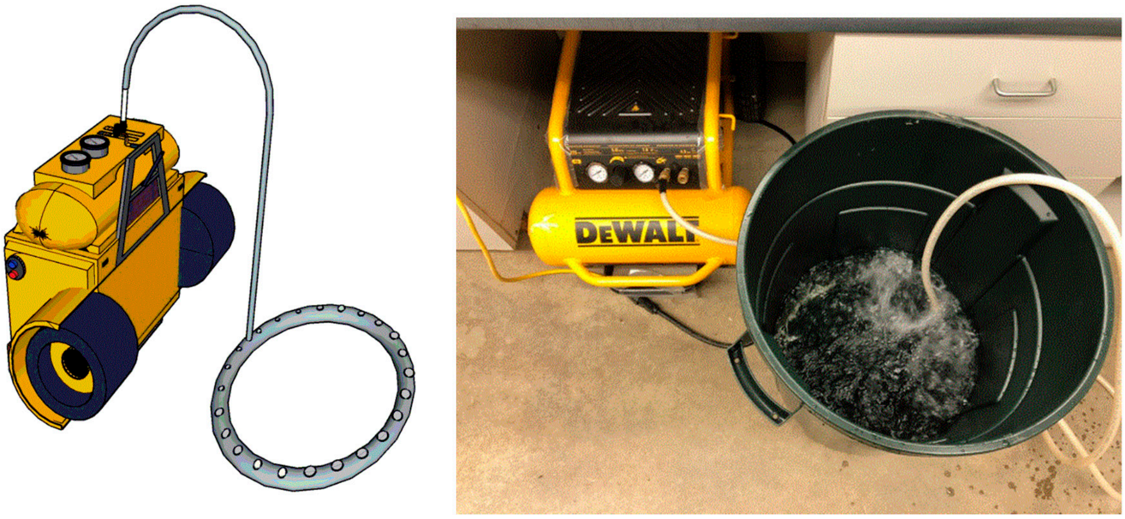

Figure S1. Generation of gaseous emissions from swine manure. Compressed air is fed into the bottom of the swine manure storage vessel. Gaseous emissions from the vessel's headspace are then blended with clean air.

Supplementary Material 2. The UV light intensity of different UV wavelength lamps

In this study, the percent reduction of target gas was investigated using two UV lights (UV-A and UV-C) with 4 different wavelengths. In the case of a UV-A LED lamp, the light intensity was measured in the 365 nm wavelength. And among the UV-C lamps, the excimer (222 nm) lamp and fluorescent (254 nm) lamp were detected at 222, 254, and 365 nm wavelengths. And only the UV-C lamp (185+254 nm) was detected at all 4 different wavelengths.

The light intensity measured depends, as expected, on the number of lamps in the chamber of the mobile lab. The measured intensities for 365 nm irradiation are given in Supplementary Table 1. Detailed measured light intensity measurements have been reported in the previous study (Lee et al., 2021).

Table S1. Measurement of UV-A light intensity according to the number of lamps in the chamber of the mobile laboratory.

Number of lamps	Light intensity (mW/cm^2)	
	Irradiated on the attached TiO_2 coating surface (photocatalysis)	Irradiated to the air inside the chamber (photolysis)
5	0.04 ± 0.04	0.06 ± 0.14
20	0.07 ± 0.06	0.40 ± 0.34
30	0.14 ± 0.12	0.59 ± 0.32
40	0.26 ± 0.23	0.70 ± 0.34
55	0.41 ± 0.33	0.88 ± 0.45

The light intensity of UV-C lamps inside chamber #2 was measured at 4 different UV wavelengths (185, 222, 254, and 365 nm). The measured intensities are reported in Supplementary Tables 2 through 7.

Table S2. The measured light intensity ($\mu W/cm^2$) with 254 nm lamp irradiation in chamber #2.

Wavelength	Location	X	Y	Z	X'	Y'	Z'	Average	S.D.
365 nm	Top	2.21	0.42	$0.55{\cdot}10^{-2}$	0.34	0.23	$0.41{\cdot}10^{-2}$	0.54	0.83
	Middle	18.7	0.44	0.61	0.33	0.61	0.14	3.47	7.46
	Bottom	1.53	0.26	$0.17{\cdot}10^{-1}$	0.26	0.30	$0.31{\cdot}10^{-2}$	0.40	0.57
254 nm	Top	8.17	0.53	0.08	1.52	1.57	0.12	2.00	3.09
	Middle	42.1	3.14	0.97	2.31	2.81	0.71	8.68	16.4
	Bottom	6.52	0.67	0.05	1.22	0.98	0.05	1.58	2.47

Table S3. The measured light intensity ($\mu W/cm^2$) of photolysis with UV-C excimer (222 nm) irradiation in chamber #2.

Wavelength	Location	X	Y	Z	X'	Y'	Z'	Average	S.D.
365 nm	Top	1.07	0.18	$0.20{\cdot}10^{-3}$	$0.31{\cdot}10^{-2}$	0.21	$0.10{\cdot}10^{-3}$	0.24	0.42
	Middle	8.91	8.10	$0.23{\cdot}10^{-1}$	1.15	7.70	$0.35{\cdot}10^{-1}$	4.32	4.33
	Bottom	2.56	0.52	$0.71{\cdot}10^{-3}$	0.31	0.38	$0.30{\cdot}10^{-3}$	0.63	0.97
222 nm	Top	7.65	2.98	0.01	0.45	2.16	0.02	2.21	2.93
	Middle	42.7	31.2	1.54	11.6	25.5	1.09	18.9	16.9
	Bottom	14.2	5.82	0.04	1.32	4.67	0.04	4.35	5.40

Table S4. The measured light intensity (μW/cm^2) with UV-C fluorescent (185+254 nm) irradiation in chamber #2.

Wavelength	Location	X	Y	Z	X'	Y'	Z'	Average	S.D.
365 nm	Top	2.23	0.45	0.40·10^{-3}	0.98·10^{-1}	0.25	0.01	0.51	0.86
	Middle	16.1	0.41	0.33	0.21	0.44	0.21	2.95	6.44
	Bottom	3.01	0.36	0.20·10^{-3}	0.10	0.39	0.66·10^{-2}	0.65	1.17
254 nm	Top	8.32	0.87	0.09	0.22	0.48	0.41	1.73	3.24
	Middle	48.2	1.38	1.42	0.79	2.73	1.02	9.26	19.1
	Bottom	6.23	0.72	0.23	0.14	0.64	0.09	1.34	2.41
185 nm	Top	0.07·10^{-1}	0.05·10^{-2}	0.02·10^{-2}	0.01·10^{-2}	0.03·10^{-2}	0.01·10^{-2}	0.01·10^{-1}	0.02·10^{-1}
	Middle	0.55·10^{-1}	0.01	0.03·10^{-2}	0.03·10^{-2}	0.03	0.03·10^{-2}	0.16·10^{-1}	0.02
	Bottom	0.04·10^{-1}	0.04·10^{-2}	0.01·10^{-2}	0.01·10^{-2}	0.02·10^{-2}	0.01·10^{-2}	0.01·10^{-1}	0.02·10^{-1}

Table S5. UV-C fluorescent (254 nm) light intensity (μW/cm^2) at 11 panels in #2 chamber (Top, Bottom, Front Top, Front Bottom, Left Top, Left Bottom, Right Top, Right Bottom, Back Top, Back Middle, and Back Bottom;).

Wave-length	T	B	FT	FB	RT	RB	LT	LB	BT	BM	BB	Aver-age	S.D.
365 nm	0.21·10^{-1}	0.15·10^{-1}	0.08·10^{-2}	0.04·10^{-2}	0.14	0.08	0.24	0.11	0.15·10^{-1}	0.04	0.21·10^{-1}	0.62·10^{-1}	0.75·10^{-1}
254 nm	0.08	0.75·10^{-1}	0.16·10^{-2}	0.21·10^{-2}	0.88	0.97	1.12	0.84	0.32·10^{-1}	0.33·10^{-1}	0.24·10^{-1}	0.37	0.47

Table S6. UV-C excimer (222 nm) light intensity (μW/cm^2) at 11 panels in #2 chamber (Top, Bottom, Front Top, Front Bottom, Left Top, Left Bottom, Right Top, Right Bottom, Back Top, Back Middle, and Back Bottom).

Wave-length	T	B	FT	FB	RT	RB	LT	LB	BT	BM	BB	Aver-age	S.D.
365 nm	0.82·10^{-2}	0.67·10^{-2}	0.02·10^{-2}	0.02·10^{-2}	0.89·10^{-1}	0.77·10^{-1}	0.82·10^{-1}	0.79·10^{-1}	0.87·10^{-2}	0.83·10^{-2}	0.78·10^{-2}	0.33·10^{-1}	0.39·10^{-1}
222 nm	0.71·10^{-1}	0.57·10^{-1}	0.11·10^{-1}	0.08·10^{-1}	1.42	1.65	1.82	1.35	0.31·10^{-1}	0.28·10^{-1}	0.03	0.59	0.78

Table S7. UV-C fluorescent (185+254 nm) light intensity (μW/cm²) at 11 panels in #2 chamber (Top, Bottom, Front Top, Front Bottom, Left Top, Left Bottom, Right Top, Right Bottom, Back Top, Back Middle, and Back Bottom).

Wavelength	T	B	FT	FB	RT	RB	LT	LB	BT	BM	BB	Average	S.D.
365 nm	$0.12\cdot10^{-1}$	$0.14\cdot10^{-1}$	$0.62\cdot10^{-3}$	$0.57\cdot10^{-3}$	$0.96\cdot10^{-1}$	0.11	0.14	0.13	$0.12\cdot10^{-1}$	$0.18\cdot10^{-1}$	$0.1\cdot10^{-1}1$	$0.49\cdot10^{-1}$	$0.56\cdot10^{-1}$
254 nm	$0.67\cdot10^{-1}$	$0.42\cdot10^{-1}$	$0.11\cdot10^{-2}$	$0.14\cdot10^{-2}$	0.63	0.72	0.72	0.74	$0.32\cdot10^{-1}$	$0.37\cdot10^{-1}$	$0.31\cdot10^{-1}$	0.28	0.34
185 nm	$0.14\cdot10^{-2}$	$0.02\cdot10^{-1}$	$0.76\cdot10^{-3}$	$0.61\cdot10^{-3}$	$0.22\cdot10^{-1}$	$0.13\cdot10^{-1}$	$0.17\cdot10^{-1}$	$0.13\cdot10^{-1}$	$0.01\cdot10^{-1}$	$0.01\cdot10^{-1}$	$0.01\cdot10^{-1}$	$0.66\cdot10^{-2}$	$0.80\cdot10^{-2}$

Table S8. Mitigation of CH_4 with the different UV doses (1.3, 2.0, 2.5, 3.9, and 5.8 mJ/cm²) irradiating gaseous emissions from swine manure.

**UV dose (mJ/cm²)**	**Light intensity (mW·cm⁻²)**	**Treatment time (s)**	**Control (ppm)**	**Treatment (ppm)**	**% reduction (*p*-value)**
UV dose control with light intensity					
1.3	0.14	9.5	1.8 ± 0.1	1.8 ± 0.1	4.7 (0.29)
2.5	0.26	9.5	1.8 ± 0.1	1.6 ± 0.1	12.9 (0.17)
3.9	0.41	9.5	2.4 ± 0.7	2.6 ± 0.4	-8.9 (0.67)
UV dose control with treatment time					
1.3	0.41	3.2	1.6 ± 0.1	1.8 ± 0.1	-13.8 (0.27)
2.0	0.41	4.8	2.8 ± 0.3	2.8 ± 0.7	0.2 (0.98)
UV dose control with light intensity & treatment time					
5.8 *	0.41 & 0.04	9.5 & 47.6	2.8 ± 0.2	2.8 ± 0.7	2.9 (0.81)

Note: * Irradiation with 5 UV-A lamps per each chamber (#1-#12, a total of 60 lamps turned on) with additional 100 portable UV-A lamps in chambers #2 and #3 turned on, 160 lamps total (installed and portable). Inlet air temperature = 19 ± 2 °C, outlet air temperature = 22 ± 5 °C.

Table S9. Mitigation of CO_2 with the different UV doses (1.3, 2.0, 2.5, 3.9, and 5.8 mJ/cm^2) irradiating gaseous emissions from swine manure. **Bold** signifies statistical significance.

UV dose (mJ/cm^2)	Light intensity (mW/cm^2)	Treatment time (s)	Control (ppm)	Treatment (ppm)	% reduction (p-value)
UV dose control with light intensity					
1.3	0.14	9.5	446 ± 29	480 ± 68	-7.5 (0.29)
2.5	0.26	9.5	446 ± 29	526 ± 57	-17.5 (0.23)
3.9	0.41	9.5	442 ± 49	532 ± 58	**-25.8 (0.01)**
UV dose control with treatment time					
1.3	0.41	3.2	598 ± 89	559 ± 61	6.6 (0.23)
2.0	0.41	4.8	463 ± 32	568 ± 89	-22.8 (0.27)
UV dose control with light intensity & treatment time					
5.8 *	0.41 & 0.04	9.5 & 47.6	478 ± 55	575 ± 56	-20.2 (0.08)

Note: * Irradiation with 5 UV-A lamps per each chamber (#1-#12, a total of 60 lamps turned on) with additional 100 portable UV-A lamps in chambers #2 and #3 turned on, 160 lamps total (installed and portable). Inlet air temperature = 19 ± 2 °C, outlet air temperature = 22 ± 5 °C.

Table S10. Mitigation of CO_2 concentration with different UV wavelength irradiating gaseous emissions inside the #2 chamber. **Bold** signifies statistical significance.

UV Wavelengths	UV dose (μJ/cm^2)	Light intensity (μW/cm^2)	Control (ppm)	Treatment (ppm)	% reduction (p-value)
185 + 254 nm	0.03	0.01	527 ± 20	771 ± 82	**-46.4 (0.04)**
222 nm	2.83	0.59		658 ± 130	-25 (0.22)
254 nm	1.78	0.37		608 ± 57	-15.5 (0.07)
367 nm	192	40		540 ± 91	-2.6 (0.80)
	1,968	410		746 ± 45	**-41.6 (0.01)**

Note: Treatment time = 4.8 s (airflow = 0.25 m^3/s), Inlet air temperature = 16 ± 1 °C, outlet air temperature = 19 ± 2 °C.
